# APOEε4 Genotype Is Related to Brain Amyloid Among Mexican Americans in the HABS-HD Study

**DOI:** 10.3389/fneur.2022.834685

**Published:** 2022-06-15

**Authors:** Sid E. O'Bryant, Melissa Petersen, James Hall, Leigh Johnson

**Affiliations:** ^1^Institute for Translational Research, University of North Texas Health Science Center, Fort Worth, TX, United States; ^2^Department of Family Medicine, University of North Texas Health Science Center, Fort Worth, TX, United States; ^3^Department of Pharmacology and Neuroscience, University of North Texas Health Science Center, Fort Worth, TX, United States

**Keywords:** Alzheimer's disease, APOE, amyloid PET, health disparities, Mexican American

## Abstract

**Introduction:**

Despite the fact that Hispanics are expected to experience the greatest increase in Alzheimer's disease (AD) and related dementias (ADRDs) by 2060, very little data is available regarding the fundamental biomarkers of AD among Mexican Americans who reflect the majority of Hispanics in the U.S. Here we sought to examine the link between *APOE*ε4 genotype and brain amyloid among Mexican Americans as compared to non-Hispanic white participants from the Health & Aging Brain Study – Health Disparities (HABS-HD) cohort.

**Methods:**

PET amyloid (florbetaben) data were analyzed from 105 Mexican American and 150 non-Hispanic white participants.

**Results:**

Among Mexican Americans, *APOE*ε4 genotype presence was associated with Global SUVR (*p* = 0.003) as well as amyloid burden in the frontal (*p* < 0.001), lateral parietal (*p* = 0.003), lateral temporal (*p* = 0.008) and anterior-posterior cingulate (*p* = 0.005) regions of interest (ROIs). Among non-Hispanic white participants, *APOE*ε4 genotype presence was associated with Global SUVR (*p* < 0.001) as well as amyloid burden in the frontal (*p* < 0.001), lateral parietal (*p* < 0.001), lateral temporal (*p* < 0.001) and anterior-posterior cingulate (*p* < 0.001) regions of interest (ROIs). The association between *APOE*ε4 genotype and cerebral amyloid was strongest among non-Hispanic white participants.

**Discussion/Conclusion:**

Despite the fact that the *APOE*ε4 genotype is significantly less frequent among Mexican Americans, its presence remains to be a significant risk factor among this group for AD pathological burden across all regions. Additional work is needed to understand the presence, progression, and clinical impact of brain amyloid among Mexican Americans.

## Introduction

Alzheimer's disease (AD) is the most common neurodegenerative disease, which disproportionately impacts underserved communities (1). In fact, Hispanics/Latinos in the U.S. (65% of which are Mexican Americans) are expected to experience the largest increase in AD and AD-related dementias (ADRDs) by 2060 (2). However, the reasons for this health disparity remain largely unknown, which is likely largely due to the substantial underrepresentation of Hispanics in AD research (3).

The 2018 AT(N) (amyloid, tau, neurodegeneration) research framework (4) provided the first ever biological-based system for studying AD and is the underlying foundation for many clinical trials and therapeutic pipelines (both academic and industry) (5). The presence of the AT(N) framework represents a tremendous advancement for the field; however, as noted in the framework publication itself, there is a significant need to examine AT(N) defined biomarkers among diverse and community-based populations (4). The representation of ethnically and racially diverse populations in research that advanced the AT(N) framework is incredibly sparse (3). Therefore, the applicability of the AT(N) defined biomarkers to the largest racial/ethnic minority groups in the U.S. remains unknown. As is the case for biomarkers in oncology (6–8), data suggests that the prevalence of pathological AD biomarkers vary by race and ethnicity (9–12).

Amyloid presence is required for the biological classification of AD-spectrum disease according to the AT(N) framework. The implications of this remains uncertain among diverse populations given that emerging data demonstrates that amyloid positivity rates may vary by race/ethnicity. Deters (13) examined data from 3,689 non-Hispanic white participants and 144 African American participants from the Anti-Amyloid in Asymptomatic AD (A4) trial and found that amyloid positivity levels were lower among African Americans as compared to the non-Hispanic white participants. In the IDEAS clinic-based dementia study, amyloid positivity rates were lower among African Americans (*n* = 639) and Hispanics (*n* = 848) as compared to non-Hispanic white participants (*n* = 15,568) regardless of diagnosis (14). In the GERAS-US Cohort study, Hispanics had lower rates of amyloid positive status (15). In our preliminary data from the HABS-HD study, amyloid positivity rates were lower among Mexican Americans as compared to non-Hispanic white participants across all cognitive diagnostic groups (16). Therefore, many clinically diagnosed AD individuals using established clinical criteria (17, 18) may result in assignment of AD that will not agree with biological assignment if that data were available. Given that there is an FDA cleared amyloid removing drug available clinically, which does not require biomarker verification for administration to patients, this means that patients from underserved populations may be incorrectly administered this medication. Therefore, there is an urgent need to understand brain amyloid among diverse populations.

The APOEε4 genotype is the single strongest genetic risk factor for late onset AD; however, a substantial literature demonstrates how the frequency of this genotype varies across populations. Corbo and Schacchi demonstrated in 1999 that APOEε4 frequencies vary significantly across the globe (19). In our prior work, we demonstrated that the APOEε4 genotype was less frequent among Mexican Americans as compared to non-Hispanic white participants (20, 21), which was cross-validated by Campos (22). Additionally, Mexican Americans have been shown to have lower APOEε4 frequency as compared to other Hispanic/Latino populations in the SOL/INCA study (23). Despite the lower frequency, APOEε4 presence may still convey significant risk for AD and associated pathology; however, this topic has not been studied among Mexican Americans. Therefore, in this pilot study, we examined the impact of APOEε4 presence on brain amyloid burden among Mexican Americans in the HABS-HD study.

## Methods

### Participants and Assessment

Data was analyzed from *n* = 105 Mexican American and *n* = 150 non-Hispanic white participants from the Health & Aging Brain Study – Health Disparities (HABS-HD; formally the Health & Aging Brain study among Latino Elders, HABLE study) that had undergone PET amyloid scans. HABS-HD study is an ongoing, longitudinal, community-based project examining health disparities in MCI and AD among Hispanic, Mexican Americans as compared to non-Hispanic white participants (16, 24–26) with recent expansion and enrollment of African Americans. HABS-HD methods have been published elsewhere (16) and are briefly outlined below. The data included in this study encompasses Mexican American and non-Hispanic white participants since the recruitment of the African American participants is ongoing. Inclusion criteria for the study includes (1) self-reported ethnicity of African American, Mexican American or non-Hispanic white participants, (2) willingness to provide blood samples, (3) capable of undergoing neuroimaging studies, (4) age 50 and above, and (5) fluent in English or Spanish. Exclusion criteria includes (1) Type 1 diabetes, (2) presence of active infection, (3) current/recent (12 month) cancer (other than skin cancer), (4) current severe mental illness that could impact cognition (other than depression), (5) recent (12 months) traumatic brain injury with loss of consciousness, (6) current/recent alcohol/substance abuse, and (7) active severe medical condition that could impact cognition (e.g., end stage renal failure, chronic heart failure, chronic obstructive pulmonary disease). Participant recruitment for HABS-HD includes a community-based participatory research (CBPR) approach (27). The CBPR approach has been used successful as a recruitment modality for reaching underserved and minority populations. It involves collaborating with local communities through outreach (holding community events, seminars), word of mouth, marketing modalities (newspaper, television, radio), and providing back information (clinical lab work, MRI clinical reads, neuropsychological test results) to the participants and their health care providers. All aspects of the study protocol can be conducted in Spanish or English. The HABS-HD study is conducted under IRB approved protocols and each participant (or his/her legal representative) signs written informed consent. All HABS-HD data is available to the scientific community through the UNTHSC Institute for Translational Research (ITR) website (28).

### Interview and Neuropsychological Assessment

An interview is conducted as part of the HABS-HD protocol, which includes an interview and neuropsychological testing with the following battery: Mini Mental Status Exam (MMSE) (29), Wechsler Memory Scale- Third Edition (WMS-III) Digit Span and Logical Memory (30), Digit Symbol Substitution, Trail Making Test Parts A and B (31), Spanish-English Verbal Learning Test (SEVLT) (32), Animal Naming (semantic fluency) (32), FAS (phonemic fluency) (33) as well as the American National Adult Reading Test (English-speakers) (34), and Word Accentuation Test (Spanish-speakers) (35). An informant interview is also conducted for completion of the Clinical Dementia Rating (CDR) Scale (4) by clinicians with expertise in dementia to evaluate for functional declines.

### Diagnostic Classification

Cognitive diagnoses were assigned algorithmically (decision tree) and verified at consensus review as follows: *C*ognitively Unimpaired (CU) = no cognitive complaints, CDR sum of boxes score of 0 and cognitive tests scores broadly within normal limits (i.e., performance greater than that defined as meeting diagnostic criteria for MCI [i.e. <=1.5 standard deviations below the normative range]); Mild Cognitive Impairment (MCI): cognitive complaint (self or other), CDR sum of boxes score between 0.5 and 2.0 and at least one cognitive test score falling <=1.5 standard deviation below normative ranges; Dementia: CDR sum of boxes score ≥2.5 and at least two cognitive test scores 2 standard deviation below normative ranges.

APOEε4 genotyping was performed using commercially available TaqMan Genotyping Kits for rs429158 and rs7412 using TaqMan GTXpress Master Mix (ThermoFisher). Target amplification and detection was performed using the 7,500 Real-Time PCR System (Applied Biosystems). Genotypes were called according to combined of allele amplification results at the two SNPs as follows (rs429358, rs7412): ε2/ε2- T,T; ε2/ε3- T,CT; ε2/ε4- CT,CT; ε3/ε3- T/C; ε3/ε4- CT,C; ε4/ε4- C,C. Positive controls (individuals of known, independently typed APOE genotypes) and negative controls were included on all runs. APOE genotypes frequencies were confirmed to be in Hardy-Weinberg equilibrium.

### PET Amyloid (Neuraceq; aka Florbetaben)

PET amyloid was conducted using florbetaben, Neuraceq) with Siemens Biograph Vision 450 whole-body PET/CT scanner following the ADNI3 protocols. Briefly, participants are injected with an 8.1 mCi (±10%) bolus of Neuraceq. A 4-frame by 5-min (20 min total) dynamic emission acquisition is started 90 min post injection following the acquisition of a low dose CT scan used for attenuation correction. The emission images are processed by iterative reconstruction, four iterations and 16 subsets. FreeSurfer-defined regions (frontal, anterior/posterior cingulate, lateral parietal, lateral temporal cortex) were used to derive a summary cortical ROI. Normalization to whole cerebellum reference region was conducted to obtain global standardized update value ratios (SUVR). An SUVR of 1.08 was used to define positivity.

### Statistical Analyses

Statistical Analyses were conducted in SPSS 25 (IBM). Chi-square and ANOVA were utilized to compare groups on demographic and clinical variables. Linear regressions were used to examine the link between APOEε4 genotype and amyloid SUVR values including age, gender, education and cognitive diagnosis (i.e., cognitively unimpaired, mild cognitive impairment, dementia) as covariates. Analyses were conducted split by ethnicity and statistical significance was set at *p* < 0.05.

## Results

Demographic characteristics of the sample are provided in Table 1. Mexican Americans were significantly younger and achieved significantly fewer years of formal education (*p* < 0.001 for both). Mexican Americans were more likely to be female (*p* = 0.02) and had significantly lower MMSE scores (*p* < 0.001). Mexican Americans and non-Hispanic white participants did not differ with regards to CDR Sum of Boxes scores. While raw SUVR levels were significantly different between Mexican Americans and non-Hispanic white participants in Global (*p* = 0.02), Frontal (*p* = 0.01), Anterior/Posterior Cingute (*p* = 0.02), and Lateral Temporal (*p* = 0.02) regions, none of these differences were significant after covarying for significant demographic differences of age, gender and education.

**Table 1:** Characteristics of sample.

	**Mexican American *n* = 105**	**Non-Hispanic white participants *n* = 150**	**Statistic**	***p*-value**
Age	67.54 (8.19)	71.80 (7.35)	*F* = 19.06	<0.001
Gender (% male)	31%	45%	χ^2^ = 5.24	0.02
Education	9.98 (4.78)	15.85 (2.36)	*F* = 168.49	<0.001
MMSE	26.34 (3.47)	28.26 (2.75)	*F* = 22.18	<0.001
CDR Sum of Boxes	0.70 (1.39)	0.55 (1.63)	*F* = 0.51	>0.05
Global SUVR	1.04 (0.14)	1.09 (0.19)	*F* = 0.85	>0.05^a^
Frontal SUVR	1.03 (0.15)	1.09 (0.21)	*F* = 1.06	>0.05^a^
Anterior/Posterior SUVR	1.10 (0.16)	1.16 (0.22)	*F* = 0.71	>0.05^a^
Lateral Parietal SUVR	1.05 (0.15)	1.08 (0.19)	*F* = 0.31	>0.05^a^
Lateral Temporal SUVR	0.99 (0.13)	1.04 (0.17)	*F* = 1.49	>0.05^a^

*SUVR, standardized uptake ratio value; MMSE, mini mental status examination; ^a^age, gender and education entered as covariates*.

The link between APOEε4 presence and cerebral amyloid is presented in Table 2 by ethnicity with the amount of variance accounted for specifically by APOEε4 genotype (covariates include age, gender, education and cognitive diagnosis). Among Mexican Americans, APOEε4 was significantly associated with global SUVR (*F* = 3.03, *p* = 0.003, 8% variance accounted for) as well as SUVR levels in the Frontal (*F* = 3.19, *p* < 0.001, 8% variance accounted for), Cingulate (*F* = 2.85, *p* = 0.005, 7% variance accounted for), Lateral/Parietal (*F* = 3.06, *p* = 0.003, 8% variance accounted for), and Lateral Temporal (*F* = 2.73, *p* = 0.008, 6% variance accounted for) regions. Among non-Hispanic white participants, APOEε4 genotype was significantly associated with Global SUVR (*F* = 4.62, *p* < 0.001, 13% variance accounted for) as well as SUVR levels within the Frontal (*F* = 4.72, *p* < 0.001, 13% variance accounted for), Cingulate (*F* = 4.22, *p* < 0.001, 11% variance accounted for), Lateral/Parietal (*F* = 4.22, *p* < 0.001, 11% variance accounted for) and Lateral Temporal (*F* = 4.09, *p* < 0.001, 10% variance accounted for) regions.

**Table 2:** APOEε4genotype and cerebral amyloid.

	**Mexican American** ***n* = 105**	**Non-Hispanic white participants *n* = 150**
Global SUVR	*F* = 3.03, *p* = 0.003 8% variance	*F* = 4.62, *p* <0.001 13% variance
Frontal SUVR	*F* = 3.19, *p* <0.001 8% variance	*F* = 4.72, *p* <0.001 13% variance
Cingulate SUVR	*F* = 2.85, *p* = 0.005 7% variance	*F* = 4.94, *p* <0.001 14% variance
Lateral/parietal SUVR	*F* = 3.06, *p* = 0.003 8% variance	*F* = 4.22, *p* <0.001 11% variance
Lateral temporal	*F* = 2.73, *p* = 0.008 6% variance	*F* = 4.09, *p* <0.001 10% variance

A total of 22 Mexican American participants were amyloid positive with a global SUVR cut-score of 1.08 whereas 40 non-Hispanic white participants were positive. Among Mexican Americans, APOEε4 was a significant predictor of amyloid positivity (OR = 6.56, 95% CI 1.95 – 22.08, *p* = 0.002) after covarying for age, gender, education and cognitive diagnosis. Among non-Hispanic white participants, APOEε4 was a significant predictor of amyloid positivity (OR = 4.67, 95% CI 1.89–11.53, *p* < 0.001) after covarying for age, gender, education and cognitive diagnosis.

Finally, ANCOVA analyses were conducted to determine if APOEε4 was associated with significantly higher cerebral amyloid levels (covariates included age, gender, education and cognitive diagnosis). Among Mexican Americans, APOEε4 was associated with significantly higher Global SUVR (1.02 vs. 1.12, *F* = 9.20, *p* = 0.003) as well as SUVRs in the Frontal (1.01 vs. 1.11, *F* = 10.19, *p* = 0.002), Cingulate (1.07 vs. 1.18, *F* = 8.13, *p* = 0.005), Lateral Parietal (1.02 vs 1.13, *F* = 9.38, *p* = 0.003), and Lateral Temporal (0.97 vs. 1.05, *F* = 7.44, *p* = 0.008) ROIs. Among non-Hispanic white participants, APOEε4 was associated with significantly higher Global SUVR (1.04 vs. 1.20, F = 21.21, *p* < 0.001) as well as SUVRs in the Frontal (1.03 vs 1.21, *F* = 22.30, p < 0.001), Cingulate (1.09 vs. 1.28, *F* = 24.37, *p* < 0.001), Lateral Parietal (1.03 vs. 1.18, *F* = 17.85, *p* < 0.001) and Lateral Temporal (1.00 vs. 1.13, *F* = 16.72, *p* < 0.001) ROIs.

## Discussion

The current work provides important additional information to the current literature. Our team has demonstrated that significant differences exist with regards to biomarkers of AD among Mexican Americans as compared to non-Hispanic white participants (16, 24–26, 36). In fact, we have shown that Mexican Americans develop cognitive loss (16) and neurodegeneration (36) at significantly younger ages when compared to non-Hispanic white participants. However, this younger age of onset is within the context of lower amyloid positivity rates (16) and lower APOEε4 genotype prevalence (20, 21). The current work demonstrates that, despite lower APOEε4 prevalence, this genetic factor remains a significant risk factor for AD-defined pathology when present among Mexican Americans.

The link between APOEε4 presence and brain amyloid among Hispanics has been very limited. Palta et al. examined the link between APOEε4 presence and brain amyloid PET among 249 community-based middle-aged Hispanics from New York City and found that APOEε4 presence was associated with significantly increased risk for amyloid positivity (15.3%) when compared to APOEε4 non-carriers (1.8%) (37). Therefore, the current findings are consistent with those from Palta et al. (37). In the current study, APOEε4 was associated with cerebral amyloid burden across regions among both Mexican Americans and non-Hispanic white participants and the amount of variance accounted for by APOEε4 genotype was consistently higher among non-Hispanic white participants. APOEε4 genotype was associated with significantly increased risk of amyloid positivity among both Mexican Americans and non-Hispanic white participants. Finally, among both Mexican Americans and non-Hispanic white participants, APOEε4 was associated with significantly higher levels of cerebral amyloid across regions. Therefore, while APOEε4 genotype, and cerebral amyloid positivity, are lower among Mexican Americans, when present, this genotype is significantly associated with worse AD-defined cerebral amyloid pathology. While the effect appears to be lower among Mexican Americans, it remains significant.

There are weaknesses to the current study. First, the sample size is relatively small; however, this is one of the only studies to specifically examine brain amyloid among Mexican Americans. Additionally, the entire HABS-HD cohort is currently undergoing brain amyloid scans and, therefore, large-scale follow-up studies will soon be underway. Another weakness is the cross-sectional nature of the data; however, HABS-HD is a longitudinal study and therefore, follow-up studies will be conducted in the future. In spite of these limitations, this is, to our knowledge, one of the only and largest studies to specifically examine the impact of APOEε4 genotype on brain amyloid levels among Mexican Americans. When placed in the context of other findings, the current findings suggest that, despite the lower frequency of APOEε4, and amyloid positivity, among Mexican Americans, the APOEε4 genotype remains a significant risk factor for AD pathology when present. Our work also points to the notion of a lower prevalence of AD-defined cognitive loss and dementia among Mexican Americans, which requires further investigation.

## Data Availability Statement

The datasets presented in this study can be found in online repositories. The names of the repository/repositories and accession number(s) can be found: http://unthsc.edu/itr.

## Ethics Statement

This study protocol was reviewed and approved by the UNTHSC IRB protocols UNTHSC 2016-128; 2020-125. Each participant (or his/her legal representative) signed written informed consent to participate in the study.

## Member of the HABS-HD Study Team Consortium

*HABS-HD Study Team: MPIs: Sid E. O'Bryant, Kristine Yaffe, Arthur Toga, Robert Rissman, & Leigh Johnson; and the HABS-HD Investigators: Meredith Braskie, Kevin King, Matthew Borzage, James R Hall, Melissa Petersen, Raymond Palmer, Robert Barber, Yonggang Shi, Fan Zhang, Rajesh Nandy, Roderick McColl, David Mason, Bradley Christian, Nicole Philips and Stephanie Large.

## Author Contributions

SEO, MP, JH, and LJ contributed to conception and design of the study. LJ organized the database. SEO performed statistical analyses. SEO, MP, JH, and LJ wrote the manuscript. SEO and LJ secured funding. All authors contributed to manuscript revision, read and approved the submitted version.

## Funding

Research reported here was supported by the National Institute on Aging of the National Institutes of Health under Award Numbers R01AG054073 and R01AG058533. This work was also supported in part by NIH/NIBIB award P41-EB015992. The content is solely the responsibility of the authors and does not necessarily represent the official views of the National Institutes of Health.

## Author Disclaimer

The content is solely the responsibility of the authors and does not necessarily represent the official views of the National Institutes of Health.

## Conflict of Interest

SEO has multiple patents on precision medicine for neurodegenerative diseases and is the founding scientist of Cx Precision Medicine. The remaining authors declare that the research was conducted in the absence of any commercial or financial relationships that could be construed as a potential conflict of interest.

## Publisher's Note

All claims expressed in this article are solely those of the authors and do not necessarily represent those of their affiliated organizations, or those of the publisher, the editors and the reviewers. Any product that may be evaluated in this article, or claim that may be made by its manufacturer, is not guaranteed or endorsed by the publisher.
